# The energy-spectrum of bicompatible sequences

**DOI:** 10.1186/s13015-021-00187-4

**Published:** 2021-06-01

**Authors:** Fenix W. Huang, Christopher L. Barrett, Christian M. Reidys

**Affiliations:** 1 Biocomplexity Institute & Initiative, University of Virginia, 995 Research Park Blvd., Suite 400, Charlottesville, VA 22911 USA; 2 Department of Computer Science, University of Virginia, Charlottesville, VA 22911 USA; 3 Department of Mathematics, University of Virginia, Charlottesville, VA 22904 USA

**Keywords:** Riboswitch, Evolutionary transition, Topological nerve

## Abstract

**Background:**

Genotype-phenotype maps provide a meaningful filtration of sequence space and RNA secondary structures are particular such phenotypes. Compatible sequences, which satisfy the base-pairing constraints of a given RNA structure, play an important role in the context of neutral evolution. Sequences that are simultaneously compatible with two given structures (bicompatible sequences), are beacons in phenotypic transitions, induced by erroneously replicating populations of RNA sequences. RNA riboswitches, which are capable of expressing two distinct secondary structures without changing the underlying sequence, are one example of bicompatible sequences in living organisms.

**Results:**

We present a full loop energy model Boltzmann sampler of bicompatible sequences for pairs of structures. The sequence sampler employs a dynamic programming routine whose time complexity is polynomial when assuming the maximum number of exposed vertices, $\kappa$, is a constant. The parameter $\kappa$ depends on the two structures and can be very large. We introduce a novel topological framework encapsulating the relations between loops that sheds light on the understanding of $\kappa$. Based on this framework, we give an algorithm to sample sequences with minimum $\kappa$ on a particular topologically classified case as well as giving hints to the solution in the other cases. As a result, we utilize our sequence sampler to study some established riboswitches.

**Conclusion:**

Our analysis of riboswitch sequences shows that a pair of structures needs to satisfy key properties in order to facilitate phenotypic transitions and that pairs of random structures are unlikely to do so. Our analysis observes a distinct signature of riboswitch sequences, suggesting a new criterion for identifying native sequences and sequences subjected to evolutionary pressure. Our free software is available at: https://github.com/FenixHuang667/Bifold.

## Background

### Bicompatible sequences in evolution

RNA evolution has been studied extensively in the framework of theoretical evolutionary optimization, center staging the genotype-phenotype mapping from RNA sequences to their structures [1–7]. RNA secondary structures are particular such phenotypes, contact structures that can be represented as diagrams, where vertices are nucleotides and arcs are base pairs drawn in the upper half-plane. If the arcs are not crossing, the represented RNA secondary structure is pseudoknot-free [8, 9]. These pseudoknot-free structures correspond to tree structures based on the lengths of contiguous subsequences. As a result, finding a structure with minimum free energy (mfe) to a given sequence can be computed efficiently using dynamic programming (DP) routines [10, 11]. Diagrams containing crossing arcs represent RNA pseudoknots [12, 13]. These pseudoknot structures can not be decomposed into trees in a straightforward fashion. Recently, pseudoknots have been studied from a topological perspective and their structural complexity was characterized by the topological genus [14–17]. In our paper, we are interested in RNA riboswitches, a small segment of an mRNA molecule that regulates mRNA translation and can express two alternative secondary structures. As riboswitches are small ($<500$ nts), they are not likely to exhibit pseudoknots [13, 18, 19]. In the following we restrict our analysis to pseudoknot-free secondary structures.

In [4], the authors realized that genotype–phenotype mappings provide a natural filtration of the sequence space by means of considering sequences “equivalent” if they fold into the same mfe-secondary structure. This perspective naturally leads to consider the induced subgraphs of preimages of the folding map in sequence space, i.e., the *neutral networks* of RNA secondary structures [5]. Neutral network are graphs, consisting of all sequences that fold into a distinguished structure, with edges connecting two such sequences if they differ in exactly one nucleotide. These networks provide a framework to quantify well-known evolutionary theories, such as Motoo Kimura’s neutral theory of evolution.

A plethora of work has been done on the diffusion-like process of sequences searching for an optimal structure, ranging from simulation-based studies [20] to the mathematical analysis of the cluster-size distribution depending on the structure of the neutral net [5]. These studies have shown that connectivity and density of neutral networks are of central importance for the understanding of how sequences evolve.

One prominent phenomenon is that of spontaneous, rapid transitions of evolving populations of RNA sequences from one structure to another—even in absence of fitness advantages [6, 21]. Despite of the Intersection Theorem [5], guaranteeing the existence of bicompatible sequences for *any* two RNA secondary structures, transitions between neutral networks are only observed for certain structure pairs. In [21], the authors showed that in the course of a phenotypic transition an evolving population of RNA sequences tunnels through bicompatible sequences. These sequences constitute de facto a gateway between different phenotypes.

**Fig. 1:**
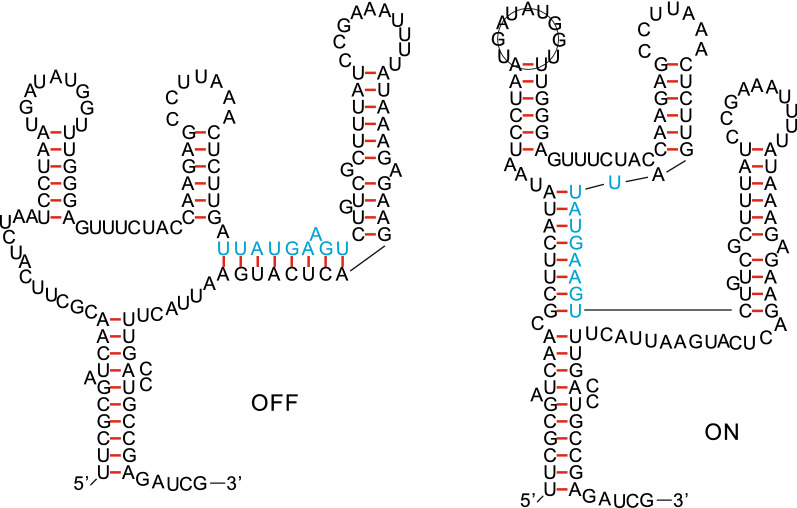
Riboswitch. Alternative structures of the Adenine riboswitch [24] and its switching sequence (blue), involved two respective helices

Bicompatible sequences play furthermore a prominent role in the analysis of RNA riboswitches [22]. Riboswitch sequences express two distinct structures, each of which appearing in a specific biophysical contexts, see Fig. 1. Both structures are typically thermodynamically suboptimal and exhibit a large base pair distance [23]. Specific mechanisms are observed, most prominently that of the existence of a *switching sequence*, a contiguous subsequence that engages for each respective structure in a unique fashion. The two structures are mutually exclusive, since bases of the switching sequence pair downstream in one and upstream in the other configuration [22].

### Finding bicompatible sequences for two structures

The studies of bicompatible sequences of a pair of secondary structures of the same length, or in more general cases where a sequence satisfies multiple structure constraints, are motivated by the computational design of RNA sequences [25–29]. Early developments such as [11, 25] design a sequence by simulation approaches. These entail considering an objective function that involves the energy contribution of multiple target structures on a common sequence. They then replicate a sequence by introducing a single random mutation, and a sequence survives if it improves the performance of the designated objective function. Later developments such as [26, 27] generate sequences satisfying multiple structure constraints based on a multi-objective genetic algorithm.

Recently, new approaches such as [30–33], design sequences on a single secondary structure using a Boltzmann sequence sampler based on the “dual” partition function of with respect to McCaskill’s partition function of secondary structures for a fixed sequence [34]. These approaches consider a sequence and a structure as a pair, and the pair can be mapped via

1 $$\begin{aligned} \varepsilon :{\mathcal {Q}}_4^n \times {\mathcal {S}}_n \longrightarrow {\mathbb {R}}^+,\quad \varepsilon (\sigma ,S)=e^{-\frac{\eta (\sigma ,S)}{KT}}. \end{aligned}$$

Here, $\eta$ is the energy function of a sequence-structure pair $(\sigma ,S)$, ${\mathbb {Q}}_4^n$ and ${\mathbb {S}}_n$ are the collections of all sequences and secondary structures of length *n* respectively, *K* is a constant(the Boltzmann constant), and *T* is a temperature parameter. The Eq. 1 resembles the notion of a scalar product of vector spaces: $V \times V \rightarrow K$, whence the notion of dual partition function. The partition function for a fixed structure induces a probability space with Boltzmann distribution where a sequence has a higher probability if it is energetically more stable with respect to the given structure.

For multiple structures, [35, 36] made use of a combination of graph coloring theory and heuristic local optimization in order to find sequences whose energy landscapes are dominated by the prescribed conformations. Later development in [28] presents an algorithm to sample sequences with multiple structure constraints with uniform probability.

In [29], the authors developed a Boltzmann sampler to generate sequences for two secondary structures of the same length such that the total energy of the two sequence-structure pairs is minimized. Their sampler is based on a simplified energy model focusing on the energy contribution of helices, while the energy contributions from hairpin loops, interior loops, and multi-loops are ignored. The key challenge is to decompose the loops contained in the two structures in a tree structure such that a DP-routine can be employed to compute their partition function. In their approach, a hyper-graph model is used to describe the intersection relationships among the loops. Assume a tree decomposition of the hyper-graph is given with tree-width *w*, then the time complexity of computing the partition function is $O(4^{w+1} n)$, thus fixed-parameter tractable (FPT). However, in the general case, finding a tree decomposition of a hyper-graph with minimum tree-width is NP-hard. Bodlaender’s famed result [37] shows that checking whether a tree decomposition with tree-width $\le k$ exists and constructing a tree decomposition with tree-width *k*, if such *k* exists, can be performed in linear time. However, it remains unknown how big the gap between such *k* and the minimum tree-width is. As the sampler in [29] considers only simple loops formed by helices, the induced hyper-graph is usually simple, whence an optimal tree decomposition can be approximated. Though the authors in [29] claim their approach can be generalized to a full-loop energy model [38], the hyper-graph will become more involved, and an optimal tree decomposition is arguably no longer easy to obtain.

### Boltzmann sampling

**Fig. 2:**
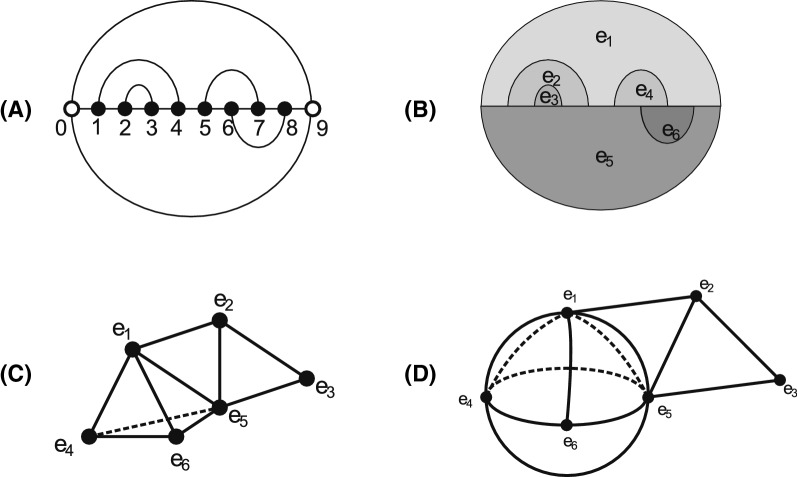
From two secondary structures to a topological space: **A** two secondary structures drawn in the upper- and lower-half plan respectively. A rainbow arc (0, 9) is added to both of the structures to close to exterior loop. **B** The secondary structures are decomposed into loops. The loops induce a hyper-graph $G=(V=\{0,\ldots , 9\},E)$, where $E=\{e_1,e_2,e_3,e_4,e_5, e_6\}$ with $e_1=\{0,1,4,5,7,8,9\},\,e_2=\{1,2,3,4\},\, e_3=\{2,3\},\,e_4=\{5,6,7\}$, $\,e_5=\{0,1,2,3,4,5,6,8,9\}$ and $e_6=\{6,7,8\}$ are loops. **C** The nerve over *E*. A $(d-1)$-simplex represent the nontrivial intersection of *d* loops. **D** The topological quotient space *K*(*R*) induced by **C**, where a ribbon induced by $\{e_1, e_2, e_5, e_3\}$ is glued to a sphere induced by $\{e_1, e_5, e_4, e_6 \}$

In order to understand how the time complexity is affected when considering a full-loop energy model [38], we express the loop intersection relationship by means of a novel topological framework [39]. The framework considers each loop as a vertex (a 0-simplex), and *d* loops having nontrivial intersection as $a (d-1)$-simplex. Thus, the simplicial complex obtained by gluing all *d*-simplices, for all $d\ge 0$, gives rise to a topological space. The simplicial complex is called a *loop nerve*, see Fig. 2. Specifically, the 0-simplices representing the loops are equivalent to the hyper-edges in the hyper-graph model discussed in [29], while the *d*-simplices for $d>0$ capture additional information that is not present in the hyper-graph model.

**Fig. 3:**
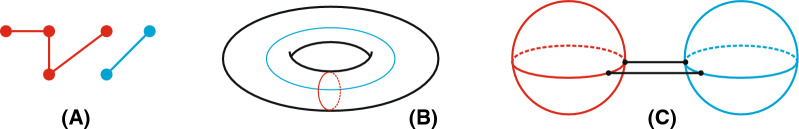
Geometric interpretation of the 0th-, 1st-, and 2nd- homology group. **A** The rank of the 0th homology group is 2, counting the number of connected components, where each of them can be contracted to a single point. **B** The rank of the 1st homology group is 2, counting the number of uncontactable circles on a torus. **C** The rank of the 2nd homology group is 2, counting the number of empty volume (2-dimensional holes) induced by the spheres

We observe that the time complexity of computing the partition function is closely related to the structure of the topological space, in particular whether or not it contains *d*-dimensional holes. In topology, *d*-dimensional holes are elements of the *d*th homology group and the latter constitute a key signature of the space. The easiest way to understand the significance of this is to consider a disc and a punctured disc. While the former can continuously be contracted into a point, the latter does not allow for such a contraction. We illustrate the geometric interpretation of the 0th-, 1st-, and 2nd-homology group in Fig. 3. Homological signatures play a central role in the context of differentiating topological spaces, since any homeomorphism of topological spaces induces an isomorphism of homologies. For instance, the characterization of closed surfaces as a sphere, a finite connected sums of projective planes, or a finite connected sums of tori uses the fact that the homologies of spheres, projective planes and tori are distinct.

In [39] it is shown that the loop nerve induced by a pair of secondary structures has an associated topological quotient space of a very particular type: it is obtained by gluing tetrahedra, spheres and ribbons. It turns out that the tetrahedra can be processed via homotopies and the resulting space may be depicted as an apple tree, the spheres representing the apples and the ribbons the branches. In the absence of spheres, we shall obtain an optimal algorithm to compute the partition function of a pair of secondary structures, i.e., we can obtain in this case a tree decomposition of the hyper-graph in [29] with minimal tree-width. It is the spheres, that are responsible for the computational complexity and an optimal polynomial time algorithm is at present unknown.

The topological framework introduced does, at present, not solve the NP-hardness rooted in the tree decomposition with minimum tree-width, it does however present a fundamentally different approach to the study of computational complexity problems associated with computing mfe-sequences to two or more secondary structures. One might speculate that simplicial complexes specifically the recently developed framework of weighted complexes (a generalization in which simplices are endowed with a certain weight) may prove useful for larger classes of computational complexity problems.

The framework presented here is tailored to express intrinsic relations between loops of different structures and reveals a detailed blueprint of how they organize, namely as topological wedge-sums of spheres.

It is well-known [40, 41] that even identical genotypes can lead to many different phenotypes in response to environmental changes, and riboswitches are natural sequences that can easily access a variety of phenotypes. Phenotypic accessibility is closely connected to bicompatible sequences and we shall employ the sequence sampler for two secondary structures in a full-loop energy model [38] to investigate properties of RNA riboswitch sequences on multiple levels. First, we compare the energy spectra of sample sequences under the constraint of being compatible and bicompatible, respectively. We show that for the two alternative structures of a riboswitch, the energy spectra of compatible sequences with either one structure is similar to the sequences that are bicompatible, i.e. compatible with both structures. This implies that riboswitch sequences can switch between their assumed structures easily, without affecting the free energy since there exist multiple, energy favorable bicompatible sequences. In contrast, random structure pairs do not facilitate such phenotypic switches, i.e. native riboswitches are distinguished sequences.

Second, we analyze how sequence-structure pairs rank within the partition function by comparing riboswitch sequences with random sequences. The rank analysis allows us to conclude that the relative rank of native riboswitch sequences is distinctively higher than the relative rank of the sampled sequences. Accordingly, native sequence exhibit an optimized thermodynamic stability with respect to the pair of structures.

Thirdly, we investigate a certain adaptability index, measuring the capability of a structure *R* to change into the structure *S*. We find that the adaptability of the alternative structures of riboswitches are distinctively different from that of random structure pairs. This indicates that the two alternative structures of a riboswitch are more evolutionary accessible by sequences than random structure pairs.

## Materials and methods

### Bistructures

**Fig. 4:**
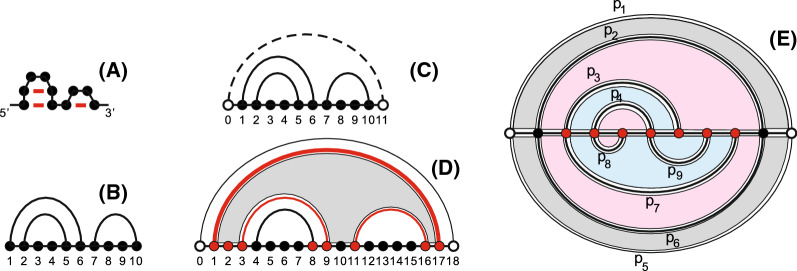
**A** An RNA secondary structure. **B** Diagram representation of **A**. **C** A secondary structure with a rainbow arc (dash). **D** Loops in a secondary structure. The loop (gray) contains a distinguished maximal arc (1, 17). **E** A bistructure *B* is a collection of loops $\{L_1, \ldots , L_9\}$. $X = \{L_3, L_7, L_9\}$ (blue) is an irreducible substructure of *B* with its complement ${\overline{X}} = \{L_1, L_2, L_4, L_5, L_6, L_8\}$. We mark the exposed vertices $E^X = V^X \cap V^{{\overline{X}}}$ in red. The closure of *X* is given by ${\tilde{X}} = \{L_2, L_3, L_4, L_6, L_7, L_8, L_9\}$

We present an RNA secondary structure as an arc diagram, a graph whose vertices are drawn on a horizontal line and the Watson-Crick as well as Wobble base pairs are drawn as arcs in the upper half-plane [42–44], see Fig. 4B. The vertices are labeled by $V=\{1,2,\ldots , n\}$ from left to right, representing the nucleotides. The linear order of the vertices indicates the direction of the RNA strand from $5'$-end to $3'$-end. Here we consider only the canonical Watson-Crick and Wobble base pairs in an RNA secondary structure. As a result, for any pair of nucleotides, there can be at most one such canonical base pair, each vertex can be only incident to one arc.

An arc, (*i*, *j*), represents the base pair between the *i*th and *j*th nucleotides. Two arcs (*i*, *j*) and (*r*, *s*) are called crossing if and only if $i<r<j<s$ holds. An RNA structure is called a secondary structure, if it does not contain any crossing arcs. Furthermore, the arcs of a secondary structure can be endowed with the partial order: $(r,s) \prec (i,j)$ if and only if $i<r<s<j$. We shall introduce two “formal” vertices associated with positions 0 and $(n+1)$, respectively and add the formal arc $(0, n + 1)$, referred to as the *rainbow*. An *interval*, [*i*, *j*], is the set of vertices $\{i,i+1,\ldots ,j-1,j\}$.

In a loop-based energy model [38, 45], arcs and unpaired vertices are organized in *loops* contributing to the energy. A loop, *L*, is a subset of vertices, represented as a disjoint union of *S*-intervals, $L={\dot{\bigcup }}_{i=1}^k [a_i,b_i]$, such that $(a_1,b_k)$ and $(b_i,a_{i+1})$, for $1\le i\le k-1$, are arcs (including the rainbow arc $(0, n+1)$) and where any other interval-vertices are unpaired, see Fig. 4D. It can be represented by a maximal arc $(a_1,b_k)$ with respect to the partial order $\prec$. Given a loop, this maximal arc is unique, whence a loop can be represented by $L_{(a_1, b_k)}$. In particular, the rainbow arc, $(0,n+1)$, represents an exterior loop, that is not nested in any arc in the arc diagram, see Fig. 4C. Furthermore, each non-rainbow arc appears in exactly two loops, being maximal for exactly one of them. Loops correspond to the boundary components of the secondary structure viewed as a fatgraph [46]. In the following, we shall identify loops with their sets of vertices.

Given two secondary structures, *R* and *S*, having the same vertex set $V=\{1,\dots ,n\}$, we draw the vertices on a horizontal line, the arcs of *R* in the upper and the arcs of *S* in the lower half-plane. We refer to this arc diagram as a *bistructure*, *B*(*R*, *S*). The idea of considering a secondary structure pair of given length has been studied in [47, 48]. Here we shall distinguish the *R*-arcs from the *S*-arcs even though they might have the exact same endpoints. For example an arc (*i*, *j*) in *R* is denoted by $(i,j)_R$ and an arc (*i*, *j*) in *S* is denoted by $(i,j)_S$. In a loop-based model, the *R*-loops and the *S*-loops are distinct since their represented *R*-arcs from the *S*-arcs are distinct. Hence, a bistructure *B*(*R*, *S*) can be considered as the set of loops $B = \{L_{p_i} \mid p_i \in B(R,S), 1\le i \le m\}$, where $p_i$, $1\le i \le m$, is an arc in *B*(*R*, *S*), see Fig. 4E.

A *substructure* of *B*, denoted by $B'$, is a subset of loops where $B' \subseteq B$. The vertex set of $B'$, denoted by $V^{B'}$, is the union of vertices in loops that are contained in $B'$. The complement of $B'$, ${\overline{B'}} = B \setminus B'$, with its vertex set $\overline{V^{B'}}$, see Fig. 4. Accordingly, we have (a) $V^{B'}\cup \overline{V^{B'}}$ contains all vertices in *B*(*R*, *S*) and (b) $V^{B'}\cap \overline{V^{B'}}$ is not necessarily empty, since paired and unpaired vertices can be contained in the intersection of the $B'$- and ${\overline{B'}}$-loops. Furthermore, for a given substructure $X=\{L_1,\cdots ,L_k\}$, we define the boundary of *X* by $X^C = \{ L\in {\overline{X}}| \exists L_i\in X, L\cap L_i\ne \varnothing \}$, i.e., $X^C$ is the set of all loops in the complement of *X* that have nontrivial intersection with *X*. We call ${\tilde{X}} = X\cup X^C$ the *closure* of *X*. A substructure is called *reducible* if the loop set can be bi-partitioned into two sets of loops $X_1 = \{L_{i_1}, \ldots , L_{i_m}\}$ and $X_2 = \{L_{j_1}, \ldots , L_{j_n}\}$, such that $L_{i_t} \cap L_{j_s} =\varnothing$, $\forall 1\le t \le m$, $1\le s \le n$, otherwise we call *X* *irreducible*, see Fig. 4E.

The intersection $E^{B'}=V^{B'}\cap \overline{V^{B'}}$ is called the set of *exposed* vertices of $B'$. The exposed vertices are key elements in computing the partition function of a bistructure, since the vertices are contained in multiple loops and their nucleotide information needs to be remembered until the energies of the loops containing the exposed vertices are calculated.

### Partition function and Boltzmann sampler

We first recall the notion of a partition function for sequences that are compatible to a single structure *R* [34].

$$ Q(R) = \sum _{\sigma \in {\mathbb {C}}_n(R)} e^{-\frac{\eta (\sigma , R)}{KT}}. $$

Here ${\mathbb {C}}_n(R)$ denotes the set of *R*-compatible sequences while $\eta (\sigma , R)$ is the energy of the sequence-structure pair $(\sigma , R)$. Lastly, *K* is the Boltzmann constant and *T* the temperature. In Turner’s model [38, 45], $\eta (\sigma , R) = \sum _{L\in R} \eta (\sigma , L)$, where *L* is a loop contained in the secondary structure *R*. The energy of a loop *L* is a function of its type and of the nucleotides associated to the arcs and the unpaired bases it contains. In practice, the energy computation takes into account a maximum of two specific arcs and four unpaired vertices, as well as the *number* of arcs and the number of unpaired bases.

For a bistructure *B*(*R*, *S*) and a sequence $\sigma$, we set $\eta (\sigma , B(R,S)) = \frac{1}{2}(\eta (\sigma ,R) + \eta (\sigma ,S))$. Then we define the partition function of sequences bicompatible to *R* and *S* by

$$ Q(R ,S) = \sum _{\sigma \in {\mathbb {C}}_n(R,S)} e^{-\frac{\eta (\sigma , B(R,S))}{KT}}, $$

where ${\mathbb {C}}_n(R,S)$ denotes the set of bicompatible sequences to both *R* and *S*.

A decomposition of *B* is a block sequential loop removal of the bistructure. Let us first illustrate the computation of *Q*(*R*, *S*) when a specific decomposition is given. Suppose $X = \{L_1,\ldots , L_k\}$ is a substructure of *B*(*R*, *S*) with vertex set *V*, and exposed vertex set $E^X$. ${\overline{X}} =B \setminus X$ denotes the complement of *X*. Let $\sigma _X = (\sigma _v)_v$ denote a subsequence with $v\in V$, $\sigma _v \in \{{{\mathbf{A}},{\mathbf{U}},{\mathbf{G}},{\mathbf{C}}}\}$. Then we can compute the energy $\eta (\sigma _X, X)$ since the nucleotide information of the vertices contained in *V* is specified. Let further $\tau _X = (\tau _v)_v$ be a subsequence where $v\in E^X$, $\tau _v \in \{{{\mathbf{A}},{\mathbf{U}},{\mathbf{G}},{\mathbf{C}}}\}$. Clearly, $\tau _X \subseteq \sigma _X$. For $\ell = |V|$, we define a partition function for *X* that is parameterized by $\tau _X$

$$ Q(X, \tau _X) = \sum _{\sigma _X \in \Sigma _\ell } e^{-\frac{\eta (\sigma _X, X)}{KT}}.$$

Here, $\Sigma _\ell$ is the collection of RNA sequences of length $\ell$.

By definition, if *X* is an irreducible substructure, then removing a loop *L* from *X* produces a set of irreducible substructures $X_1,\ldots , X_k$. We investigate how the exposed vertex set evolves with a loop removal. To this end let $x\in E^X$ be an exposed vertex. If $x\in L$, then either (a) $\not \exists L'\in X, L'\ne L$ such that $x\in L'$, or, (b) at least one such $L'$ loop exists. In the first case (a), we have *x* is no longer exposed, while in the second case (b), we have $x\in E^{X_i}$ for some $1\le i\le k$. Finally, if $x\notin L$ to begin with, then we have $x\in E^{X_i}$ for some $1\le i\le k$ after removing *L* form *X*.

Let $\tau _X$ denote a fixed subsequence over $E^X$, $\tau _{X_i}$ a subsequence over $E^{X_i}$, $1\le i \le k$, and $\sigma _L$ a subsequence over the loop *L*. We consider all possible subsequences $(\sigma _v)_v$ where $\sigma _v \in \{{{\mathbf{A}},{\mathbf{U}},{\mathbf{G}},{\mathbf{C}}}\}$, $v\in \left( L \cup _{i=1}^k E^{X_i} \right) \setminus E^X$. Then, the partition function $Q(X, \tau _X)$ can be computed recursively by

2 $$ Q(X, \tau _X) = \sum _{(\sigma _v)_v} e^{-\frac{\eta ((\sigma _L, L)}{KT}} \prod _i^k Q(X_i, \tau _{X_i}). $$

**Fig. 5:**
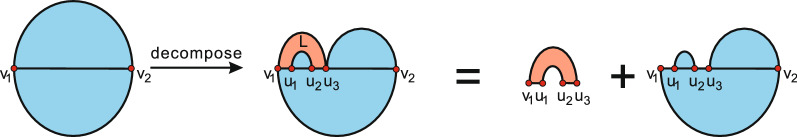
Illustration of the recursion for the partition function. Given a bistructure *X* with exposed vertices $v_1$ and $v_2$, we decomposed *X* into a loop $L(v_1, u_1, u_2, u_3)$ and a substructure $X'(v_1, u_1, u_2, u_3, v_2)$. Here we assume $v_1$, $u_1$, $u_2$, $u_3$, and $v_2$ are contained in loops of $X'$, hence $v_1$ and $v_2$ remain exposed and $u_1$, $u_2$, and $u_3$ are newly exposed vertices

For a given decomposition, the terms $Q(X_i, \tau _{X_i})$, for $1\le i \le k$, can be computed in parallel. We illustrate the recursion 2 in Fig. 5.

When *Q*(*R*, *S*) is computed, we can Boltzmann sample RNA sequences following the classical stochastic backtracking method introduced by [49], which is of linear time complexity. Given an irreducible substructure *X* that is decomposed into a loop *L* and a set of irreducible substructures $X_1, \ldots , X_k$. Assume the nucleotides in $X_i$ are sampled, then with a fixed subsequence $\tau _X$ over the exposed vertex set $E^X$, the subsequence $(\sigma _v)_v$, $v\in L \setminus \cup _i E^{X_i}$ is sampled with probability

$$ \frac{ e^{-\frac{\eta ((\sigma _v)_v, L)}{KT}} \prod _i^k Q(X_i, \tau _{X_i}) }{ Q(X, \tau _X) }. $$

Multiplying all inside probabilities of each iteration, we conclude that a sequence is sampled with probability ${\mathbb {P}}(\sigma ) = e^{-\frac{\eta (\sigma , B(R,S))}{KT}}/Q(R,S)$.

### The topology of a bistructure

The partition function *Q*(*R*, *S*) is computed recursively based on substructures, where a loop is removed from the substructure to calculate its energy. The removal yields a collection of substructures having fewer loops. In this recursion, a nucleotide $\tau _i$ at position *i* needs to be stored until the energy of all loops containing $\tau _i$ are calculated. For a fixed decomposition $D = \{X_0,\ldots , X_k\}$, the number of nucleotides that need to be stored at each step of the recursion 2 is $|L \cup _{i=1}^k E^{X_i}|$. We denote the maximum number of $|L \cup _{i=1}^k E^{X_i}|$ in all recursion steps by $\kappa _D(B)$. For a fixed decomposition *D*, $\kappa _D(B)$ is a constant.

Assume *D* is a decomposition of *B*(*R*, *S*), we can implement a dynamic programming (DP) routine to compute *Q*(*R*, *S*) recursively. The time complexity of the algorithm is $O(4^{\kappa (B)} n)$ since for every $\sigma _v$, $v\in L \cup _{i=1}^k E^{X_i}$, we have four nucleotide choices ${{\mathbf{A}},{\mathbf{U}},{\mathbf{G}},{\mathbf{C}}}$. Therefore, the algorithm to compute *Q*(*R*, *S*) is an FPT algorithm, as it can be solved in polynomial time (as a function of *n*) when assuming $\kappa (B)$ is a constant. However, $\kappa _D(B)$ can be very large, thus contributing a significant factor to the time complexity. Thus, a decomposition *D* that minimizes $\kappa _D(B)$ is desired. Let further $\kappa (B) = \min _D \kappa _D(B)$, i.e. the minimum time complexity over all possible decompositions *D*. Clearly, $\kappa (B)$ depends only on the bistructure *B*.

It is impossible to consider all decomposition since there are exponentially many of them. The algorithm introduced in [29] computed the partition function following an analogous DP-routine as Eq. 2. The key question is then to find a “smart” decomposition of the bistructure. The authors in [29] develop a hyper-graph model to interpret the overlapping relationships among all loops. In their hyper-graph model, a labeled vertex represents a nucleotide at a fixed position, and a hyper-edge represents a loop. Then a tree decomposition of the hyper-graph induces a hierarchy tree structure for the loops. Following the tree decomposition, one can derive a removal order of loops in the bistructure, and by construction the tree-width $w = \kappa _D(B) -1$.

Finding a tree decomposition with minimum tree-width for a general hyper-graph is NP-hard. However, for the simple energy model in [29], the hyper-graph may be simple enough such that a tree decomposition with minimum tree-width can be found by approximation algorithms [37]. It is not clear whether this is still feasible when passing to a full-loop energy model.

Loop intersections are studied in [39] via a simplicial complex, where a loop in a bistructure *B*(*R*, *S*) is represented by an abstract 0-simplex, i.e., a vertex. This line of work goes beyond the hyper-graph approach in that intersections of multiple loops can be consistently expressed and unlocks powerful concepts from algebraic topology. If *d* loops have nonempty mutual intersection, they are represented by a $(d-1)$-simplex. The collection of all $d\ge 0$-simplices forms a simplicial complex, called a *loop nerve*, see Fig. 2. A loop removal is tantamount to deleting the corresponding 0-simplex as well as all higher dimensional simplices that contain it. We shall show that understanding the structure of the topological space provides insight into designing an optimal decomposition.

**Fig. 6:**
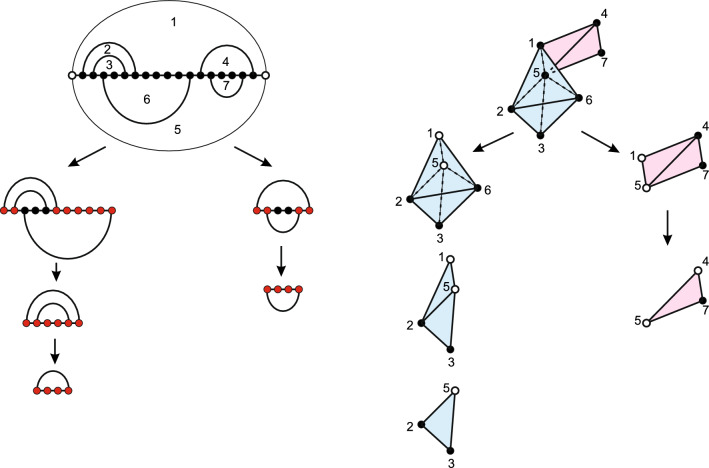
A decomposition of a bistructure (LHS) and the evolution of its loop nerve (RHS). LHS: exposed vertices are labelled red. RHS: the loops 1, 2, 3, 5 and 6 form a sphere. Removing one loop from this sphere is tantamount to deleting one vertex of the loop nerve. The white vertices in the loop nerve represent the boundary of the substructure. The sphere corresponding to the crossing component is resolved by removing the *S*-arc

We first give an overview of how to design the decomposition of a bistructure via the topological framework. In [39], the authors show that the induced loop nerve of a bistructure *B*(*R*, *S*) has very specific properties. The topological space is uniquely classified by the rank of its second homology group $r_2(B)$, which counts the number of 2-dimensional holes. As mentioned before, the geometric realization is comprised of ribbons glued to filled tetrahedra and spheres. Each sphere has a combinatorial interpretation within the bistructure as a crossing component and their number equals the rank of the second homology group. We shall show that the challenge of the decomposition problem stems from the spheres, as the ribbons and tetrahedra are organized in a tree-like fashion. The global tree-like structure induces a tree decomposition naturally, while the sphere can be resolved locally. To resolve the spheres we can map the problem to a known NP-problem such as, for instance, the traveling salesman problem (TSP). This allows us to solve the spheres via approximation algorithms [50]. We illustrate this idea in Fig. 6.

### Topological framework

In the following we discuss the results in [39].

#### **Definition 1**

Suppose *B*(*R*, *S*) is a bistructure having *n* loops $B = \{L_1,\dots ,L_n\}$. We call $Y =\{ L_{i_0}, \ldots , L_{i_d}\}$ a *d*-simplex of *B* if and only if $\bigcap _{k=0}^d L_{i_k} \ne \varnothing$. Let $K_d(B)$ be the set of all *d*-simplices of *B*. Then the nerve of *B* is

$$ K(B) = \dot{\bigcup }_{d=0}^{\infty } K_d(B)\subseteq 2^B. $$

**Fig. 7:**
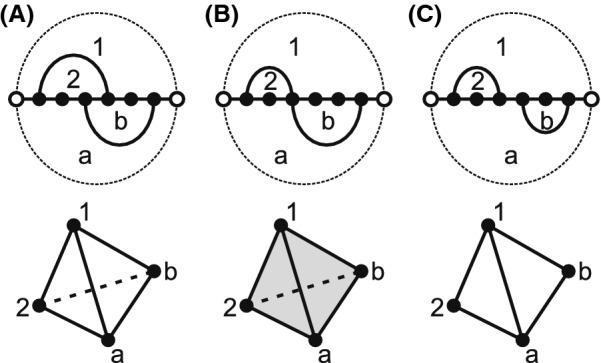
Examples of topological realizations of the loop nerves for different bistructures: **A** an empty tetrahedron or a sphere), **B** a filled tetrahedron, and **C** two filled triangles glued along a mutual edge

The loop nerve *K*(*B*) has the topological space *T*(*B*) as its quotient space [51], see Fig. 7. The 0-simplices correspond to hyper-edges in [29]. In the loop nerve the collection of *d*-simplices, encapsulates the information of loop intersections not articulated in the hyper-graph model.

The loop nerve of a bistructure *B* contains no *d*-simplex for $d>3$ [39] and there are only two nontrivial homology groups of *T*(*B*), both being free and abelian: $H_0(T(B)) \cong {\mathbb {Z}}$ and $H_2(T(B)) \cong \oplus _k^r {\mathbb {Z}}$. In view of connectivity, the rank of $H_2(T(B))$, $r_2(B)$, is the only determinant. An *R*-arc (*i*, *j*) and an *S*-arc (*r*, *s*) are called crossing if $i<r<j<s$ holds. We shall proceed by discussing overlaps and crossing components.

**Fig. 8:**
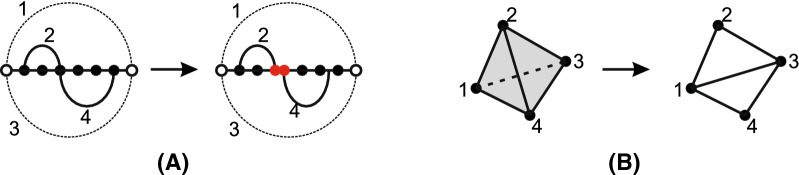
Splitting an overlap without inducing crossing arcs (**A**). The split is tantamount to removing an edge of the corresponding filled tetrahedron as well as its interior, ending up with two triangles that are still glued along the opposite edge from the edge we removed. **B** The split effect on the corresponding simplicial complex of **A**

An *overlap* is a degree four vertex in its arc diagram. An overlap corresponds to a 3-simplex in $K_3(X)$ in the loop nerve. Assume *x* is an overlap being the endpoint of the arcs $p_1 \in R$ and $p_2\in S$. We split *x* into two adjacent vertices $x_1$ and $x_2$, where $x_1$ carries the endpoint of $p_1$ and $x_2$ the endpoint of $p_2$ . This is done such that after the split $p_1$ does not cross $p_2$, see Fig. 8

We next convince ourselves that the split does not “really” affect the induced topological space, where “really” means “upto homotopy”. Let *x* be an overlap. This vertex is contained in four loops and is the endpoint of two arcs $p_1$ and $p_2$. Let $L_1$ and $L_2$ be the loops in *R* that contain $p_1$, where $p_1$ is the maximal arc of $L_2$. Furthermore let $L_3$ and $L_4$ be the loops in *S* that contain $p_2$, where $p_2$ is the maximal arc of $L_4$. Clearly, $\cap _{i=1}^4 L_i = \{x\}$. Splitting *x* into $x_1$ and $x_2$ [39] is tantamount to removing an edge of the corresponding filled tetrahedron as well as its interior. We end up with two triangles that are still glued along the opposite edge from the edge we removed, see Fig. 8. This splitting does not change $r_2(B)$, whence we can restrict ourselves to the non-overlap case.

In the arc diagram of a bistructure we adopt the notion of crossing component as in [39].

#### **Lemma 2**

*Let* $X^O$* be the substructure induced by a crossing component* *O,* *and let* $\tilde{X^O}$ *be its closure. Then the induced topological space of* $\tilde{X^O},$ $T(X^O),$ *is homeomorphic to an empty sphere, and thus contributes 1 to the rank of* $H_2(B).$

We define the $*$-graph of the loop nerve to be the graph $\Delta (B)=(K_2(B),E)$ with edges given by

$$ E\ni e=(\Delta _1,\Delta _2)\Leftrightarrow \Delta _1\cap \Delta _2\in K_1(B). $$

Each vertex in the $*$-graph represents a filled triangle in *T*(*B*), and there is an edge between two vertices if their respective triangles have nonempty intersection along an edge. Then we have:

#### **Lemma 3**

*Let* *X* *be a substructure without crossing arcs and overlaps, i.e.,* $H_2(B)=0$ *and* $K_3(B)=\varnothing.$ *Then its* *-graph $\Delta (B)$ *is a tree.*

**Fig. 9:**
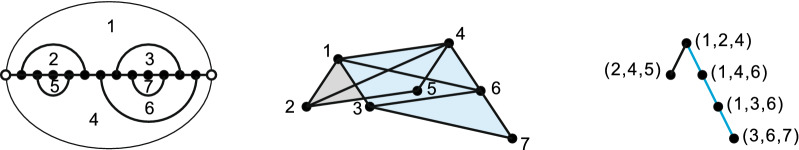
A bistructure having no overlap and crossing arcs (left), its loop nerve (middle), and the $*$-graph (right). Each vertex in the $*$-graph presents a triangle in the loop nerve, labeled by a triple of loops. Its $*$-graph is a tree

We illustrate the $*$-graph of a bistructure without overlaps and crossing arcs in Fig. 9. Note that, by Lemma 3, if *B* has no crossings, the induced topological space *T*(*B*) is a “ribbon tree”. Namely, each ribbon is obtained by gluing a sequence of triangles along their edges such that each triangle has at most two edges glued to other triangles. These ribbons are then glued together along some of the edges of their constituent triangles such that no closed bands appear.

Now we are in position to describe the structure of the topological space *T*(*B*). If an irreducible substructure *X* is induced by a crossing component, then the induced topological space is “sphere”-like. Otherwise if *X* is noncrossing, the induced topological space is “ribbon tree”-like. *T*(*B*) is a ribbon tree modulo edge contraction of spheres [39]. Finally, we have the combinatorial interpretation of $r_2(B)$ and given a bistructure *B*(*R*, *S*) with *r* crossing components. then $r_2(B) = r$.

### Scheduling

We next discuss how to design a decomposition based on the properties of the loop nerve. The global tree-like structure induces a tree decomposition naturally, while the sphere will be resolved locally. We first consider the case where *B* contains no crossing arc. In this case, we extend the partial order $$\prec $$ for a bistructure by the following: for any two arcs $$(i,j),(r,s)\in B$$ we say $$(i,j) \prec _B (r,s)$$ if and only if $$ i<r<s<j$$. Then, we show in the SM that for an irreducible substructure $$X\subseteq B$$, *X* contains a unique maximal arc with respect to $$\prec _B$$.

We decompose *X* by removing the loop $L_{m}$, where *m* is the maximal arc of *X*. The loop removal produces a set of irreducible substructure $X_1, \ldots , X_k$. Repeating this loop removal for any produced irreducible substructures gives a unique loop removal order $D_0(B)$. We show

#### **Lemma 4**

*Let* *B(R,* *S)* *be a bistructure without crossing arcs or overlaps. Let* $D_0$ *be the loop removal order discussed above. For any loop removal order* $D\ne D_0,$ *we have* $\kappa _{D_0}(B) \le \kappa _{D}(B),$ *i.e.,* $D_0$ *is a decomposition that minimizes* $\kappa (B).$

**Fig. 10:**
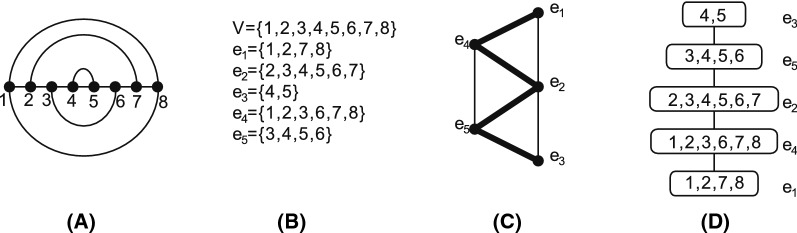
Topologically guided optimal tree decomposition: **A** a bistructure without crossing arcs or overlaps. **B** The hyper-graph of **A**. **C** Its quotient space is a ribbon. **D** The optimal tree decomposition of **B** obtained by following (**C**)

A bistructure *B* with overlaps can be mapped to a bistructure $B'$ without overlaps by the above splitting of overlapping vertices. The decomposition $D_0$ on $B'$ induces a natural decomposition *D* on *B* by the one-to-one correspondence between the *B*-arcs and the $B'$-arcs. We illustrate in Fig. 10 how to derive a decomposition of a hyper-graph with minimum tree-width for the case where *B*(*R*, *S*) is a bistructure without crossing arcs or overlaps.

We next discuss how to resolve spheres. Recall that the NP-hardness of the decomposition problem stems from the spheres. In this case, we consider mapping the problem to a known NP-problem as, for instance, the traveling salesman problem (TSP). To this end we remove a set of loops from *X* with a minimum number of exposed vertices, such that *X* has no crossing arcs. The remaining noncrossing substructure can be decomposed using the optimal algorithm presented before, see Fig. 6. This allows to solve the problem via approximation algorithms of the TSP [50]. The approximation approach is the subject of future work and beyond the scope of this paper, for the analysis presented below, we employ a greedy approach to resolve the spheres.

### Structural adaptability

In this section, we introduce three measures for quantifying the structural adaptability using the bicompatible sequence sampler. Given two structures *R* and *S*, we first sample sequences with single-compatible and bicompatible constraints respectively, and compare their energy spectrum of sampled sequences paired with *R* and *S*. In case of bicompatibility not affecting the energy spectrum significantly, we can conclude that switching from *R* to *S* (and vice versa) is feasible. Secondly, we investigate the energy ranking of $(\sigma ,R)$ and $(\sigma ,S)$, within the partition function of $\sigma$. This shows how stable *R* and *S* are in the Boltzmann ensemble of a sampled sequence. Finally, we introduce an index, called the *adaptability*, measuring the capability of a structure *R* to transform into *S*. The adaptability is obtained by comparing the proportion of the partition function for bicompatible sequences w.r.t. the partition function of single-compatible sequences. In case of all sequences being bicompatible, the index equals 1, while in the case of sequences not being bicompatible, the index equals 0.

#### Energy spectra

Let us begin by introducing the spectrum over a partition function. Let *Q*(*X*) be a partition function of sequences compatible with *X*, where *X* is a secondary or bistructure and $Q(X)=\sum _\sigma e^{-\frac{\eta (\sigma , X)}{KT}}$. To simplify notation, we shall write *Q* instead of *Q*(*X*), if we do not need to emphasize the context of the underlying structure *X*. Naturally, *Q* induces the discrete probability space $(\Sigma _n,{\mathbb {P}}_{Q})$, where ${\mathbb {P}}_{Q}(\sigma )=e^{-\frac{\eta (\sigma ,X)}{KT}} / Q$. We consider a real-valued random variable $f :(\Sigma _n,{\mathbb {P}}_{Q}) \longrightarrow {\mathbb {R}}$ and refer to the induced measure ${\mathbb {P}}_{f}$ on ${\mathbb {R}}$, ${\mathbb {P}}_{f}(r)= \sum _{\{\sigma \mid f(\sigma )=r\}} {\mathbb {P}}_{Q} (\sigma )$, as the *f*-spectrum over *Q*.

For practical purposes, an *f*-spectrum, ${\mathbb {P}}_f(r)$, cannot be computed directly, since we have to consider all $\sigma \in \Sigma _n$ and potentially infinitely many $r\in {\mathbb {R}}$. To approximate the *f*-spectrum we first discretize by means of a monotone increasing multiset $(a_s)$, where $\Delta =a_s-a_{s-1}$, setting ${\mathbb {P}}_f(a_s) = \sum _{a_{s-1}<r \le a_s} {\mathbb {P}}_f(r)$. We employ a Boltzmann sampler to generate sequences of the probability space $(Q_4^n,{\mathbb {P}}_Q)$ and approximate ${\mathbb {P}}_{f}(a_s)$ by ${\mathbb {P}}_{f}(a_s) \approx \frac{1}{m} \mid \{\sigma \mid a_{s-1} < f(\sigma ) \le a_s\} |$, where $\sigma$ is a sequence sampled from the partition function *Q* and *m* denotes the sample size. Here we set $m=10^4$.

We proceed by introducing some particular choices for the pair (*f*, *Q*), which we shall denote by $f_Q$:

$$ f^R_{Q}(\sigma )=\eta (\sigma ,R) \quad  f^S_Q(\sigma )= \eta (\sigma , S). $$

We call ${\mathbb {P}}_{f_Q^R}(r)$ the *R*-*spectrum of* *Q* and ${\mathbb {P}}_{f_Q^S}(r)$ the *S*-*spectrum of* *Q*.

#### Ranking

Next we investigate how stable *R* and *S* are in the Boltzmann ensemble of $Q(\sigma )$, where $\sigma \in (\Sigma _n,{\mathbb {P}}_{Q(R,S)})$ is a sequence sampled via *Q*(*R*, *S*). We compare the energies, $\eta (\sigma , R)$ and $\eta (\sigma , S)$ to $\eta (\sigma , M(\sigma ))$, where $M(\sigma )$ denotes the mfe-structure of $\sigma$. Then we consider the ratios

$$ r_R = \frac{\eta (\sigma , R)}{\eta (\sigma , M(\sigma ))}, \quad r_S = \frac{\eta (\sigma , S)}{\eta (\sigma , M(\sigma ))}. $$

For a fixed sequence $\sigma$, the ratios reflect the gap between the energies obtained when considered with *R* (or with *S*) and the mfe-structure.

#### Adaptability

We discuss the energy-spectrum over a partition function, *Q* as an induced measure of a random variable, *f*. By construction we normalize, when working with the probability measure ${\mathbb {P}}_{Q}(\sigma )$, the value of *Q*. As a result, the absolute values of the different partition functions, for instance, when comparing *Q*(*R*) and $Q(R)|_S$ is not a factor.

Comparing a plethora of riboswitches, as well as sequences of various random structure pairs, we end up with relating the partition functions of sequences over an entire spectrum of lengths. The free energy of any sequence is however the sum of loop energies, and each loop has a unique maximal arc. In Jin and Reidys [52] it is shown that the number of arcs in random structures satisfies a central limit theorem, whence its mean scales linearly with *n*. This implies that the number of loops grows linearly with *n*, which in turn suggests that the free energy of a sequence grows linearly with *n*.

Accordingly, we consider the scaled partition function

$$ {\tilde{Q}}(R)=\sum _{\sigma }e^{\frac{1}{n}\frac{\eta (\sigma ,R)}{KT}} \quad {\tilde{Q}}(R)|_S=\sum _{\sigma \in {\mathbb {C}}_n(S)} e^{\frac{1}{n}\frac{\eta (\sigma ,R)}{KT}} $$

and set

$$ w_R = \log \left( \frac{{\tilde{Q}}(R)|_S}{{\tilde{Q}}(R)}\right) , \quad  w_S = \log \left( \frac{{\tilde{Q}}(S)|_R}{{\tilde{Q}}(S)}\right) . $$

We call $w_R$ and $w_S$ the *densities* of the structure *R* and *S* respectively. The adaptability $w_R$ is a real number in [0, 1] measuring the proportion of the partition function of *R* composed by bicompatible sequences relative to that composed by sequences compatible only with *R*. The closer this adaptability is to 1, the more energy favorable bicompatible sequences there are, suggesting that the structure *R* can change into the structure *S* more easily. Note that by construction $w_R$ and $w_S$ are asymmetric, namely, the transitions from *R* to *S* and those from *S* to *R* are not necessarily equal.

## Results

In this section we focus on using the RNA sequence sampler to study the sequence-structure relations of RNA riboswitch sequences. The time complexity of the algorithm presented here is $O(4^{\kappa (B)} n)$, where $\kappa (B)$ is constant depending only on the bistructure *B*, and *n* is the length of the two input sequences. Currently, we can only deal reliably in case of $\kappa (B) \le 20$. In the following, we select six riboswitch sequences from the literature, across different classes, organisms and switching mechanisms, exhibiting a sequence length below 250nts, and $\kappa (B) \le 20$, see Table 1.

**Table 1:** The riboswitch sequences

Riboswitch	add_Adenine	xpt_Guanine	mtgE_Mg	lysC_Lysine	VEGFA	yitJ_SAM
Abbr	add	xpt	mgt	lys	VEGFA	sam
Length	147	162	213	243	125	170
Class	Adenine	Guanine	Magnesium	Lysine	Het. nuclear ribonucleoprotein L	*S*-Adenosylmethionine
Organism	*Vibrio vulnificus*	*Bacillus subtilis*	*Bacillus subtilis*	*Bacillus subtilis*	*Homosapiens*	*Bacillus subtilis*
Function	Translation	Transcription	Transcription	Transcription	Alt. Splicing	Transcription
Energy on (kcal/mol)	− 92.90	− 94.61	− 150.00	− 148.05	− 89.18	− 78.76
Energy off (kcal/mol)	− 99.90	− 109.80	− 135.80	− 166.56	− 55.20	− 93.70
Reference	[24]	[53]	[54]	[55]	[56]	[57]

### Energy spectra

We are now in position to study the *R*- and *S*-spectra of *Q*-Boltzmann sampled, bicompatible sequences of specific structure pairs, or equivalently the *R*- and *S*-spectra of sequences compatible to bistructures using the measure of “Energy spectra” section. It will be interesting to compare these with the spectra of the compatible sequences of each respective secondary structures, *R* and *S* and to provide a comparative analysis of the *R*- and *S*-spectra of native structures with that of random structure pairs.

Let *R*, *S* be two secondary structures, to begin the comparative analysis of compatible and bicompatible sequences we compare *Q*(*R*) and *Q*(*S*), given by

$$ Q(R) = \sum _{\sigma \in \Sigma _n} e^\frac{{-\eta (\sigma ,R) }}{KT}, \quad Q(S) = \sum _{\sigma \in \Sigma _n} e^\frac{{-\eta (\sigma ,S) }}{KT}, $$

with the partition functions

$$ Q(R)|_S = \sum _{\sigma \in {\mathbb {C}}_n(R, S)} e^\frac{{-\eta (\sigma ,R) }}{KT}, \quad Q(S)|_R = \sum _{\sigma \in {\mathbb {C}}_n(R,S)} e^\frac{{-\eta (\sigma ,S) }}{KT}. $$

*Q*(*R*) and *Q*(*S*) are recursively computed and their Boltzmann samplers are introduced in [32, 33]. On an abstract level, $$Q(R)|_S$$ and $$Q(S)|_R$$ were *a priori* available by means of rejection Boltzmann samplers for *Q*(*R*) and *Q*(*S*). However, sampling such sequences is impractical as the probability of randomly encountering a bicompatible sequence is too low. As a first application, our framework developed in “Materials and methods” section we observe that $$Q|_S(R) $$ and $$Q|_R(S)$$ can be computed by replacing the energy function $$\eta (\sigma , B(R,S)$$ by $$\eta (\sigma , R)$$ and $$\eta (\sigma , S)$$, respectively.

Comparing the *R*- and *S*-spectra of *Q*(*R*) with $Q(R)|_S$ and *Q*(*S*) with $Q(S)|_R$, respectively, allows us to draw conclusions about the difficulty of the process of modifying a *R*-compatible sequence into an *R*, *S*-bicompatible sequence, while maintaining the energy with respect to *R* and the analogue statement for *S*.

**Fig. 11:**
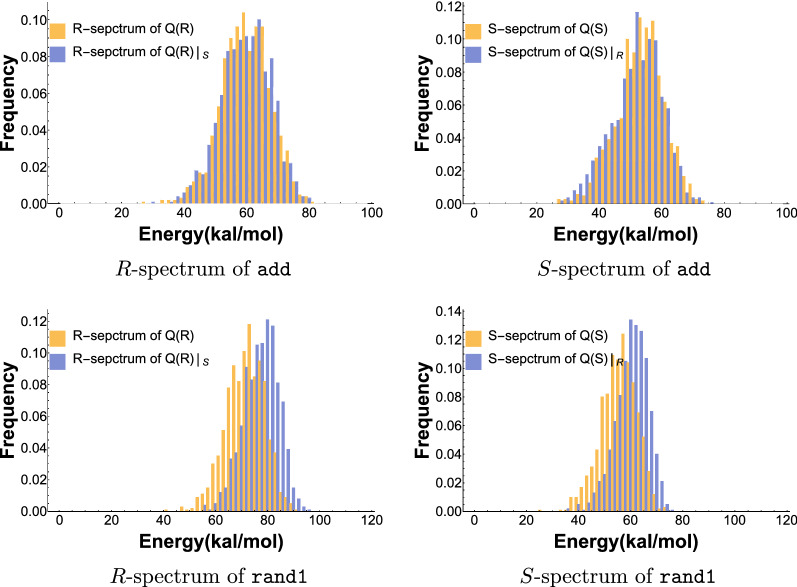
The *R*- and *S* spectra of the riboswitch structure pair, add and rand1. Top left: $f_{Q(R)}^R$ versus $f_{Q(R)|_S}^R$ for add. Top right: $f_{Q(S)}^S$ versus $f_{Q(S)|_R}^S$ for add. Bottom left: $f_{Q(R)}^R$ versus $f_{Q(R)|_S}^R$ for rand1. Bottom right: $f_{Q(S)}^S$ versus $f_{Q(S)|_R}^S$ for rand1

In Fig. 11d we compare $f_{Q(R)}^R$ with $f_{Q(R)|_S}^R$, i.e. the *R*-energy spectra over *Q*(*R*) and $Q(R)|_S$ (LHS) and $f_{Q(S)}^S$ with $f_{Q(S)|_R}^S$, i.e. the *S*-energy spectra over *Q*(*S*) and $Q(S)|_R$ (RHS). We remark that these are pairwise comparisons of measures over distinctively different, nested probability spaces.

We show that the *R*-energy spectra over *Q*(*R*) and $Q(R)|_S$ and *S*-energy spectra over *Q*(*S*) and $Q(S)|_R$ pairwise coincide. This means, that modifying a *R*-compatible sequence into a *R*, *S*-bicompatible sequence can be done without affecting the free energy with respect to *R* and vice versa for *S*. Moreover, this finding holds for all native, as well as random structure pairs we analyzed. The results for the other riboswitch as well as random structures pairs are shown in the SM.

**Fig. 12:**
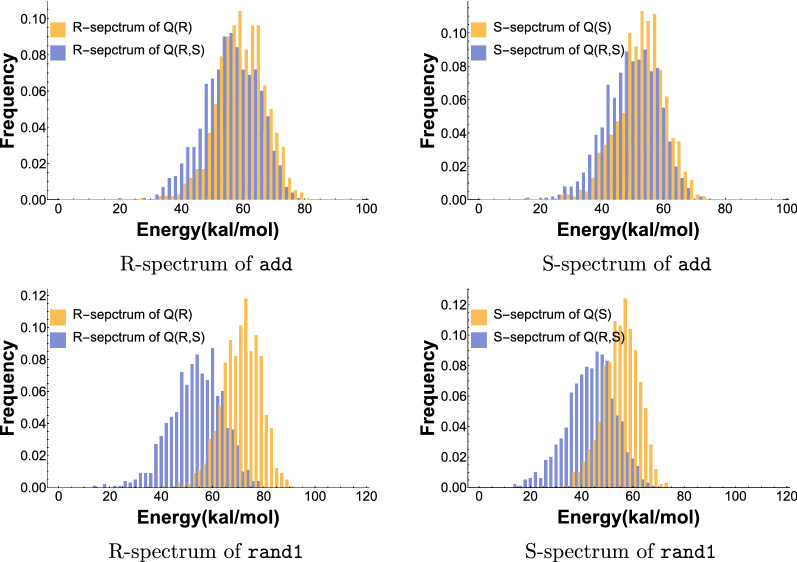
The *R*- and *S* spectra of the riboswitch structure pair, add and rand1. Top left: $f_{Q(R)}^R$ versus $f_{Q(R,S)}^R$ for add. Top right: $f_{Q(S)}^S$ versus $f_{Q(R,S)}^S$ for add. Bottom left: $f_{Q(R)}^R$ versus $f_{Q(R,S)}^R$ for rand1. Bottom right: $f_{Q(S)}^S$ versus $f_{Q(R,S)}^S$ for rand1

The next step is to relate bicompatible sequences, Boltzmann sampled from *Q*(*R*, *S*) to those Boltzmann sampled from *Q*(*R*). In the context of the $Q(R)|_S$ versus *Q*(*R*) analysis, we now factor in the energy with respect to *S*. While *R*-energy levels can be maintained while satisfying the *S* base-pairing conditions, it turns out to be much more intricate to derive bicompatible sequences that are well suited for both *R* and *S* at the same time. Thus we consider

$$ Q(R,S) = \sum _{\sigma \in {\mathbb {C}}_n(R, S)} e^\frac{{-1/2 \cdot (\eta (\sigma ,R) + \eta (\sigma ,S))}}{KT} $$

and compare the energy spectra $f_{Q(R,S)}^R$ and $f_{Q(R)}^R$ as well as $f_{Q(R,S)}^S$ and $f_{Q(S)}^S$ for a variety of riboswitch sequences and their respective native structure pairs, see Table 1. We display the energy spectrum of the riboswitch add in Fig. 12(top). The energy spectra and detailed analysis of the other riboswitch sequences is presented in the SM.

In order to put our results into context, we present an analysis of the spectra $f_{Q(R,S)}^R$ and $f_{Q(R)}^R$ as well as $f_{Q(R,S)}^S$ and $f_{Q(S)}^S$ for structure pairs that are not riboswitches. We consider two random RNA sequences and fold them to their mfe-structures, where each structure is thermodynamically stable to not only one of the sequences, but to both. For practical reasons, we consider sequences of length 150, which is close to the average length of natural riboswitches. We show a representative result for in Fig. 12(bottom). Our observations are remarkably robust: the energy spectra of random structure pairs are literally identical and we provide a detailed analysis of additional spectra in the SM.

Figure 12(top left) shows that the *R*-spectrum, $f_{Q(R,S)}^R$, is practically identical to the *R*-spectrum, $f_{Q(R)}^R$. The *S*-spectra behave completely analogous, there is no significant difference between the *S*-spectrum $f_{Q(R,S)}^S$ and $f_{Q(S)}^S$, see Fig. 12(top right). In the SM we provide additional data and the spectra of the riboswitch sequences listed in Table 1. The phenomenon holds robustly for all riboswitch sequences we analyzed.

For random structure pairs, however, the picture changes: the *R*-spectrum, $f_{Q(R,S)}^R$, is shifted distinctively to the left of the *R*-spectrum, $f_{Q(R)}^R$, see Fig. 12(bottom left). The same holds for the *S*-spectra: $f_{Q(R,S)}^S$ is shifted distinctively to the left of the *S*-spectrum $f_{Q(S)}^S$, see Fig. 12(bottom right). More data for random structure pairs are presented in the SM. The different patterns of the *R*-spectrum and *S*-spectrum for riboswitches in contrast to random structure pairs reveals a strong signal as the two structures corresponding to a riboswitch are strongly correlated. As such, the signal can be used to identify riboswitch features.

### Ranking

**Fig. 13:**
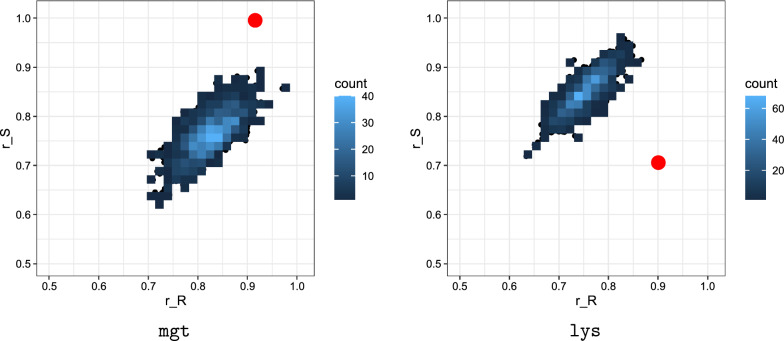
$(r_R, r_S)$ for the riboswitches mgt (left) and lys (right): Boltzmann sampled sequences versus native sequences. For each riboswitch we Boltzmann sample $10^3$ sequences and compute $(r_R, r_S)$ for the sampled sequences (blue). We contrast this with $(r_R, r_S)$ of for the respective, native riboswitch sequence (red)

We compute the $(r_R, r_S)$ for the riboswitches mht and lys according to the test introduced in “Ranking”. We display our result in Fig. 13.

Figure 13 displays the ratios $(r_R, r_S)$ for the riboswitch mgt (LHS) and lys (RHS). The figure is obtained based on the Boltzmann sampling of $10^3$ sequences from (*Q*(*R*, *S*)) (blue) and displays in addition the ratios of the native sequences of mgt and lys (red).

We find for mgt that all ratios satisfy $r_R > 70\%$ and $r_S > 62\%$ and furthermore the respective (coordinatewise) means are $(85\%, 74\%)$. For lys we have $r_R > 64\%$ and $r_S > 72\%$ with a mean of $(78\%, 85\%)$. Accordingly, *R* and *S* are suboptimal structures in the Boltzmann sampled sequences. Furthermore, we find that the ratio of ratios, $r_R/r_S$ is almost constant within the set of sampled sequences. For both, mgt and lys alike, $(r_R, r_S)$ of the native sequence is distinctively higher than the ratio pairs obtained from the sampled sequences. This indicates that the native sequence exhibits an evolved thermodynamic stability with respect to the pair of structures (*R*, *S*).

### Density

**Fig. 14:**
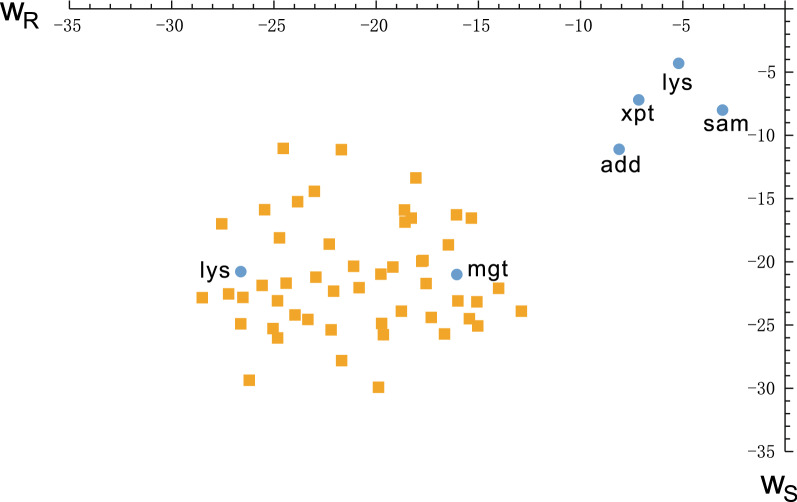
The energy weighed space of bicompatible sequences. $(w_R,w_S)$ of the six riboswitch structure pairs, presented in Table 1 (blue). Furthermore: $(w_R,w_S)$ of 50 random structure pairs of length 150, as control set (red)

We compute $(w_R,w_S)$ for all riboswitch structures pairs in Table 1 according to measure introduced in “Adaptability” section, and augment the analysis by inspecting 50 random structure pairs as control set. The random structure pairs are obtained using the method described in “Energy spectra” section. We consider random structure pairs having length of 150, which is close to the average sequence length of riboswitches. We display in Fig. 14 the pairs $(w_R,w_S)$.

Figure 14 shows that the pairs $(w_R,w_S)$ of riboswitch sequences are distinctively different from random structure pairs and appear in the top right corner, while the $(w_R,w_S)$ of random structure pairs are shifted towards the bottom left corner. The closeness of ratio pairs displayed in Fig. 14 to the top right corner represents how likely a sequence, Boltzmann sampled from ${\tilde{Q}}(R)$, is contained in ${\tilde{Q}}(R)|_S$. This reflects how dense the bicompatible sequences sampled from ${\tilde{Q}}(R)$ are within the compatible sequence Boltzmann sampled from ${\tilde{Q}}(R)$. Figure 14 shows that this adaptability is significantly higher for native riboswitch sequences, compared to sequences Boltzmann sampled from ${\tilde{Q}}(R)|_S$ for random structure pairs (*R*, *S*).

## Discussion

The time complexity of computing the partition function for a given pair of secondary structures is determined by the decomposition of the bistructure and the computation of the partition function. Hammer et al. [29] shows that when assuming a tree decomposition is given with a constant tree-width, the partition function can be computed in polynomial time. However, obtaining such a tree decomposition with minimum tree-width remains an NP-hard problem. The work in [29] focuses on the former, presenting an algorithm that computes the partition function on a simplified loop-based energy model. Although in general a hyper-graph can be decomposed into a tree-like structure having tree-width *k*, the parameter *k* is not understood in a systematic way.

Although our Boltzmann sequence sampler addresses the same topic [29], i.e. the computation of the partition function for two given structures and Boltzmann sampled sequences, our approach is fundamentally different. Instead of following the established hyper-graph approach and parameterized complexity paradigm, we summon a different framework. The loop-complex introduced here allows to differentiate the relations of loops of two distinct secondary structures since intersections can occur for two, three or even four distinct loops. The simplicial complex of [39] offers a new approach for the analysis of bistructures. The topology captures higher order information by higher dimensional simplices. In [39], the authors provide a natural classification of bistructures via the rank of their second homology group, $r_2(B)$. It further describes the topological space of a given bistructure, namely, a ribbon tree modulo the contraction of spheres. In Lemma 4 we construct in case of $r_2(B)=0$, i.e., for a ribbon tree, the optimal loop removal schedule. Our implementation is based on a full-loop energy model, whereas [29] considers a simplified energy model considering only on the energy of helices. It is thus, that a direct comparison of the results between the two implementations is not being presented.

The understanding of the topological space provides deeper insight into the design of decompositions of the bistructure. The tree-like structure is naturally a tree decomposition, while the complexity of the problem originates from the spheres. Though we have not yet obtained an explicit solution to resolve the spheres, we are considering mapping the resolution to a known NP-problem like for instance, the traveling salesman problem (TSP). Via such a mapping, efficient approximation algorithms [50] can be levied.

Therefore, we believe there is a strong connection between our loop nerve framework and the tree decomposition problem, as they essentially model the same computational problem. We will further investigate such a connection in future studies. Assuming the decomposition of a bistructure is given, the implementation of the Boltzmann sequence sampler is analogous to the algorithm in [29]. Thus, sampling sequences subject to specific constraints, and sampling sequences for multiple structures can be done generalizing the loop-complex introduced in [29]. This generalization will be studied in future work.

The filtration of sequence space by mapping sequences into their mfe-secondary structure has been a powerful tool for the analysis of evolutionary optimization [4]. This search exhibits extended periods of phenotypic neutrality separated by transition events, during which structural change manifests [2, 3, 6, 21]. Bicompatible sequences facilitate these transitions, which depend heavily on the particular choice of the two structures. Our framework allows us to quantify the adaptability of the two structures by means of the adaptability $(w_R, w_S)$, see Fig. 14. The result shows that this ratio is distinctively higher for the native structure pairs of riboswitches compared to random structure pairs. As a result transitions between native structure pairs are much easier than for random pairs. This motivates to identify what properties of the native structure pairs lead to the high adaptability of their bicompatible sequences.

Over two decades multistable sequences were analyzed [25]. These are multistable w.r.t. alternative conformations, performing different functionality by switching between their alternative structures in gene regulation and as such these sequences represent beacons in evolution. Flamm et al. [25] studies sequences that are multistable with respect to two structures, both being suboptimal by mapping the problem into a combinatorial optimization problem. The latter is then solved via an adaptive walk, initiated at a random sequence. The Boltzmann sampling of the starting sequences for the adaptive walks of [25] will likely have the same speedup effect, as the Boltzmann sampling of compatible sequences in the context of inverse folding [58].

On the level of phenotypes, our results on energy spectra and adaptability of a bistructure can clearly distinguish between riboswitch or native and random structure pairs reliably, see Figs. 12 and 14. While we are not in position to formulate criteria for designing such structure pairs, we can recognize them. As for genotypes, it is not easy to identify whether a sequence is a candidate for a riboswitch–even if the native structure pair is given. Such a sequence has to contain a specific sequence pattern, facilitating the switch from one structure to the other. Various studies are trying to give criteria for riboswitch sequences. Freyhult et al. [59] considers the Boltzmann ensemble of a sequence with base pair filtration. If there are multiple clusters of structures which have high adaptability and a fixed base pair distance to the mfe-configuration, then alternative structures are predicted. However, the analysis specifies the existence of structure clusters relative to the mfe-structure and it is not clear if they can switch. The ranking $(r_R, r_S)$ in Fig. 13 allows to identify native riboswitch *sequences* from Boltzmann sampled sequences of the native riboswitch structure pair. Thus, for riboswitches both: the phenotype pair and the genotype are distinguished. The alternative structures of riboswitches have a dense set of bicompatible sequences and the native sequence assumes a particularly low energy for the alternative configurations. The latter is displayed in Fig. 13. Boltzmann sampled sequences for riboswitch structure pairs are distinctively different from the native sequence. Thus the design of riboswitch sequences involves two types of data: the structure pair, as well as the sequence simultaneously.

Our results on riboswitches captured intrinsic phenotypic transition features. However, our analysis was focussed on RNA riboswitches. As for future work , we will undertake a systematic analysis of the performance of the bicompatible sequence sampler in the context of general phenotypic transitions. In particular transitions of rapidly evolving virus populations.
